# CAFD: Context-Aware Fault Diagnostic Scheme towards Sensor Faults Utilizing Machine Learning

**DOI:** 10.3390/s21020617

**Published:** 2021-01-17

**Authors:** Umer Saeed, Young-Doo Lee, Sana Ullah Jan, Insoo Koo

**Affiliations:** 1 School of Electrical Engineering, University of Ulsan, Ulsan 44610, Korea; umarsaeed454@mail.ulsan.ac.kr (U.S.); leeyd1004@ulsan.ac.kr (Y.-D.L.); 2 School of Computing, Engineering and Physical Sciences, University of the West of Scotland, Paisley PA1 2BE, UK; sanaullah.jan@uws.ac.uk

**Keywords:** WSN, Extra-Trees, machine learning, classification, data-driven, context-aware system, sensor faults, fault diagnosis

## Abstract

Sensors’ existence as a key component of Cyber-Physical Systems makes it susceptible to failures due to complex environments, low-quality production, and aging. When defective, sensors either stop communicating or convey incorrect information. These unsteady situations threaten the safety, economy, and reliability of a system. The objective of this study is to construct a lightweight machine learning-based fault detection and diagnostic system within the limited energy resources, memory, and computation of a Wireless Sensor Network (WSN). In this paper, a Context-Aware Fault Diagnostic (CAFD) scheme is proposed based on an ensemble learning algorithm called Extra-Trees. To evaluate the performance of the proposed scheme, a realistic WSN scenario composed of humidity and temperature sensor observations is replicated with extreme low-intensity faults. Six commonly occurring types of sensor fault are considered: drift, hard-over/bias, spike, erratic/precision degradation, stuck, and data-loss. The proposed CAFD scheme reveals the ability to accurately detect and diagnose low-intensity sensor faults in a timely manner. Moreover, the efficiency of the Extra-Trees algorithm in terms of diagnostic accuracy, F1-score, ROC-AUC, and training time is demonstrated by comparison with cutting-edge machine learning algorithms: a Support Vector Machine and a Neural Network.

## 1. Introduction

Modern technologies, particularly the Internet-of-Things (IoT) and Cyber-Physical System (CPS) merged with Artificial Intelligence (AI), play a vital role in everyday life. These advanced systems have the ability to address social challenges such as environmental sustainability and economic downfalls. In this technological era, from the evolution of autonomous vehicles, smart factories, and intelligent healthcare systems towards smart homes, the IoT and CPS have captured the utmost parts of the urban world [1,2,3,4].

However, these advanced systems are based on the integration of diverse physical objects, such as sensors. To monitor physical conditions, sensors are spatially dispersed and data are collected at base stations engendering the Wireless Sensor Network (WSN) [5]. Undeniably, sensors make our lives easier by their innumerable uses. Nevertheless, they come at a cost of being prone to failures.

**Figure 1:**
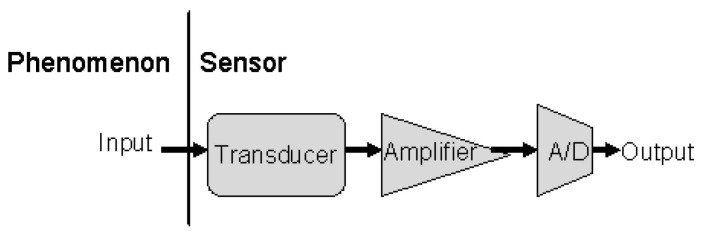
Depiction of a sensor and its key components.

Deployment of sensors in complex environments while facing natural conditions, electromagnetic interference, and other relevant factors can lead to abnormal sensor behaviors (i.e., anomalies or faults). These faults are serious threats to the economy, worker’s safety, and system’s reliability. A typical sensor, along with the data flow through major components, is shown in Figure 1. These major components trigger abnormal behavior when defective. Each component is associated with certain static-limiting properties, which can be described by specifications, and that may affect the resulting data. However, the sensor’s output can also be affected by the external environment, such as communication or battery defects, which commonly occur in a WSN [6].

To avoid any unpleasant circumstances caused by sensor failure, sensor fault detection and diagnosis has been an important area of research in recent years [7]. Generally, fault diagnostic approaches can be categorized as knowledge-based, signal-based, model-based, and hybrid techniques [8,9]. However, due to the emergence of cloud computing and Machine Learning (ML), the knowledge-based (or data-driven) approach is becoming an effective method for detection, diagnostics, and prognostics by scrutinizing the behavior of sensors through large amounts of data [10,11,12].

**Figure 2:**
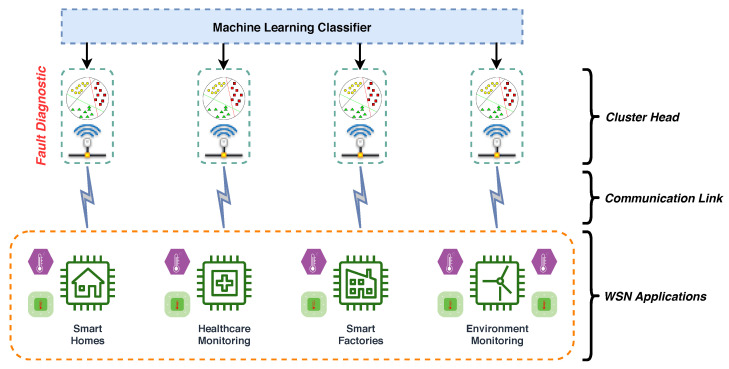
Architecture of the proposed fault diagnostic scheme.

In this work, a new Context-Aware Fault Diagnostic (CAFD) scheme towards the detection and diagnosis of faults or anomalies in sensors is employed. First, the data under consideration are multi-labeled according to the context of the fault. These data samples are then given as input to the context-based ML classifier to diagnose. Upon diagnoses (or classification), the output of the context-based classifier, along with the original data samples (or sensor output signals), are given as input to a fault-based ML classifier. Finally, the fault-based classifier categorizes the data samples in order to detect and diagnose any abnormal behavior occurring in the network. The proposed scheme is discussed in detail in Section 4, and the general architecture of the proposed system is presented in Figure 2. Multiple sensors data from distinct applications communicated to cluster heads through wireless links are shown, where a lightweight ML classifier (Extra-Trees) is deployed for timely detection and diagnosis of faults.

### Contributions

The major contributions of this paper are summarized in the following points.

- All the commonly recognized sensor faults that occur in a WSN are considered: drift, hard-over/bias, erratic/precision degradation, spike, stuck, and data-loss.
- A realistic multi-hop WSN scenario composed of humidity and temperature sensor measurements is designed with extreme low-intensity faults.
- We presented the context-aware system based on multi-label classification as well as the multi-class classification for fault diagnosis, which, to our knowledge, has not been studied in earlier research.
- Following that, the lightweight CAFD scheme is developed based on the ensemble learning algorithm called Extra-Trees to detect and diagnose sensor faults in a timely fashion.
- Lastly, the proposed CAFD scheme’s effectiveness, along with that of the Extra-Trees algorithm, is revealed by comparison with a traditional approach and advanced ML classifiers (SVM and NN). Widely employed performance evaluation metrics—diagnostic accuracy, F1-score, ROC-AUC, and training time (as lightweight measure)—are used.

The rest of this paper is organized as follows. In Section 2, related work is discussed. Section 3 presents the sensor fault taxonomy. In Section 4, the proposed CAFD scheme and classification techniques are discussed. Section 5 provides simulations and results. Finally, Section 6 concludes the paper. Acronyms and abbreviations used in this paper are listed in Table 1.

## 2. Related Work

Rapid advancement in AI techniques over the last few years has opened new doors for abnormality detection and diagnosis systems. Recently, context-aware and ensemble learning methods are widely considered due to their high performance. For instance, the authors in [13] proposed a context-aware intrusion detection system utilizing machine learning for a smart factory, and the study in [14] used an ensemble machine learning approach for disease diagnosis based on wearable sensors. The authors in [15] suggested an ensemble learning-based algorithm named Random Forest (RF) for real-time fault detection in magnetic position sensors.

In [16], the authors utilized Deep Learning (DL) techniques known as autoencoders and a Fuzzy Deep Neural Network (FDNN) for feature selection and fault diagnosis, respectively. Support Vector Machine (SVM) classification algorithm was employed to detect sensor faults, whereas the FDNN served diagnostic purposes. The authors in [17,18] exploited SVM algorithm, whereas the study in [19] employed RF to detect sensor faults, which are both well-known supervised ML classifiers. In [20], the authors proposed a Convolutional Neural Network (CNN)-based fault diagnosis method by image classification. The DL-based CNN algorithm in that work achieved considerable accuracy. The authors in [21] proposed a hierarchically fused FDNN that combines a fuzzy Neural Network (NN). In [22], a high anomaly detection rate was achieved for WSN by utilizing one-class-SVM scheme. The authors of [23] considered a Long Short-term Memory NN for fault detection and isolation against three defective classes. In earlier work [24], we proposed an ensemble learning-based lightweight approach to detect and diagnose most faults occurring in a WSN. However, a more sophisticated system that is not only lightweight but also significantly accurate is desirable.

All the aforementioned work has certain advantages and disadvantages associated with it. Some of the work is focused on detection, disregarding diagnostics. The work that focused on diagnostics did not consider all the commonly occurring faults in WSNs. In addition, the abnormality in sensors is mostly treated as a binary problem. Other than that, faults can be of extremely low intensity, for which a reliable system is required. DL techniques can be highly accurate for the feature extraction/selection and detection of low-intensity faults; nonetheless, they require high computational power for execution.

## 3. Sensor Fault Taxonomy

Faults are considered to be a divergence from normal operation in sensor output. These faults can be transient, persistent, or intermittent, depending upon the situation [25,26]. Network congestion, intricate conditions, low-quality production, and aging of the sensor are considered prime reasons for defects [27,28]. Sensor faults are categorized under the trend in which they diverge from normal operations. Mathematically, a normally operating sensor can be expressed by Equation (1):

(1) $$S_{n}=f\left(t\right)+\eta$$

where f(t) is the sensor output at time *t*, and η denotes noise. Ideally, signal $S_{n}$ will be equal to f(t), but in actual situations, a fault-free sensor will have some η associated with it. The six commonly occurring types of sensor fault considered in this research can be acquired by tampering with the above equation, which is explained as follows [8].

### 3.1. Drift Fault

In this type of commonly occurring fault, the sensor output linearly increases or decreases over time at a constant rate [29]. Let i(t) be the bias added to the output signal at time *t*, and it increases over time: i(t)=i(t−1)+α. The drift fault is modeled in Equation (2).

(2) $$\begin{array}{c} S_{n}=f\left(t\right)+\eta +i\left(t\right),\\ i(t)=i(t-1)+\alpha ,\;\alpha =constant \end{array}$$

### 3.2. Hard-Over/Bias Fault

When the sensor output shifts from normal operation to a greater state, it is called a hard-over fault. In this kind of fault, a constant rate is added to the output signal of the sensor, as modeled in Equation (3).

(3) $$\begin{array}{c} S_{n}=f\left(t\right)+\eta +\alpha ,\;\alpha =constant \end{array}$$

### 3.3. Spike Fault

As the name suggests, this type of fault is high-amplitude in an intermittent manner at constant periods in the output signal of the sensor. Let τ be the period in the sensor output at which the spikes occur. Mathematically, a spike fault is modeled in Equation (4).

(4) $$\begin{array}{c} S_{n}=f\left(t\right)+\eta +\alpha \left(t\right);\;\forall t=n\times \tau ,\\ f(t)+\eta ;\;otherwise,\;n=\{1,2,3,\ldots \},\;\tau \geq 2 \end{array}$$

### 3.4. Erratic/Precision Degradation Fault

The erratic fault adds noise to the sensor output signal, with a high variance and zero mean. This type of fault is mathematically expressed in Equation (5).

(5) $$\begin{array}{c} S_{n}=f\left(t\right)+\eta +\alpha \alpha ∼N\left(0,\delta_{\alpha }^{2}\right)\delta_{\alpha }^{2}\gg \delta_{\eta }^{2} \end{array}$$

### 3.5. Stuck Fault

In the stuck fault, sensor output undergoes zero, or nearly zero, variations. This kind of fault can be persistent as well as transient. In a persistent situation, this fault can be expressed as a complete failure. The stuck fault is modeled in Equation (6).

(6) $$\begin{array}{c} S_{n}=\alpha ,\;\alpha =constant \end{array}$$

### 3.6. Data-Loss Fault

As revealed by the name, the data-loss fault is a null state in sensor output over arbitrary time intervals. This sort of fault is primarily caused by calibration or hardware defects, and commonly occurs in a WSN.

**Figure 3:**
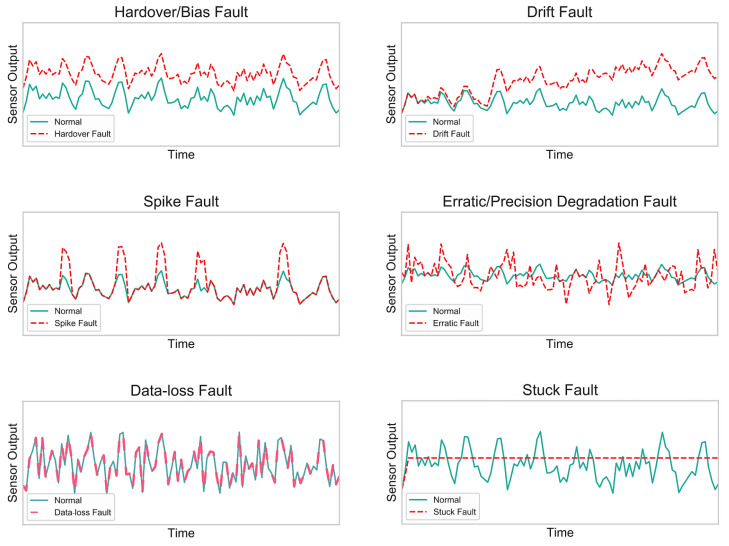
Normal/legitimate and faulty signal sample plots.

**Figure 4:**
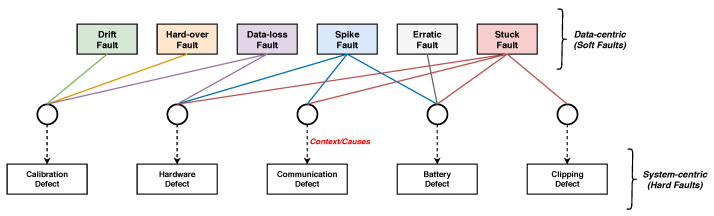
Representation of abnormal sensor behaviors (or faults) according to the context.

Figure 3 illustrates sample plots of the aforementioned faults (or abnormal behaviors) along with the normal state, whereas Figure 4 represents these faults according to their context. These contexts can be explained as the internal or external environments of the sensor that are accountable for triggering the faults. As shown in Figure 4, there can be a single cause or multiple causes for a fault to arise. For instance, drift and hard-over faults are primarily caused by a calibration defect (or error), while a data-loss fault is caused by either calibration or hardware defects. Moreover, the spike fault can appear as a consequence of hardware, communication, or battery defects. The erratic fault is the result of a battery defect, whereas a stuck fault can be triggered by several defects, such as hardware, communication, battery, and clipping.

## 4. The Proposed CAFD Scheme

### 4.1. Classification Techniques

**Figure 5:**
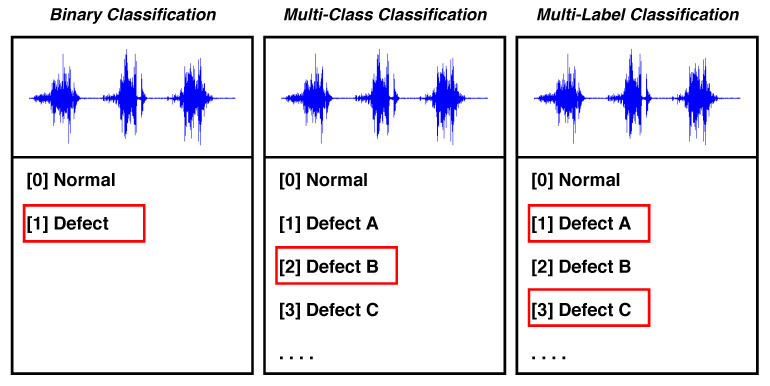
Types of classification.

As an instance of supervised learning, classification is a technique to identify data observations according to where they belong on the basis of training data. Classification is primarily divided into three categories: binary, multi-class, and multi-label. Figure 5 exemplifies the types of classification.

- **Binary Classification** involves two classes. A set of data observations (or data samples) can only be assigned to one of the two classes. For instance, in sensor fault detection, data observations are categorized as either a normal class or an abnormal class.
- **Multi-Class Classification** deals with a single target variable of the individual class. In other words, this technique comprises more than two mutually exclusive classes. Data observations are categorized into multiple classes based on disparity. For instance, in sensor fault diagnosis, data observations of multiple classes are classified into any one of the target variables, such as a normal class, a drift fault class, a hard-over fault class, and so forth.
- **Multi-Label Classification** handles multiple target variables of the individual class. This technique is employed when data observations of a class concurrently belong to two or more target variables. For instance, in the stuck sensor fault class scenario, data observations simultaneously belong to multiple target variables, such as hardware, communication, battery, and clipping (as shown in Table 2).

### 4.2. Extra-Trees

Extra-Trees (ET), commonly known as Extremely Randomized Trees, is an algorithm based on an ensemble of Decision Trees (DTs) [30]. The idea behind the ensemble technique is a combination of multiple DTs and taking decisions established by a fused network. The same flux is followed by the RF algorithm, but with two key differences. First, ET works on the principle of random splitting phenomena, whereas RF follows the best-splitting technique. Second, ET retreats data observations without any replacement (Bootstrap=False), whereas the observations are withdrawn with a replacement under RF.

Furthermore, node splitting in a tree is the process of transforming a non-homogeneous root node into homogeneous split/child nodes. The random splitting technique of ET divides the root node into random child nodes, but in RF, the best-splitting approach coverts the root node to homogeneous child nodes. The advantage of ET over RF or other ML classifiers is the high reduction of variance and bias error. High variance can cause the overfitting problem, while a high bias can provoke underfitting. Moreover, the ET ability in randomness makes it computationally faster, and robust towards noisy features [24,31].

The workflow of the ET algorithm can be explained in four key steps. First, training set *X* is given as input at the root node. Second, the algorithm randomly selects *N* samples from *X* without replacement. Third, it constructs a tree from the learning samples and, at each child node, selects *F* features randomly and splits the node into arbitrary cut-points. Fourth, the ET aggregates the outcome of each tree by repeating the second and third steps *T* times. Furthermore, the most significant parameters to be taken care of while generating the ET model are: the number of DT in the ensemble *k*, the number of features to randomly select *f*, and the minimum number of samples needed to split the node $n_{min}$.

### 4.3. System Model

The term *context* in “Context-Aware Fault Diagnostic” refers to the interior or exterior conditions (or environment) of the sensors, and *aware* relates to the conscious intelligent ML algorithm. The idea behind the CAFD system is to utilize the contexts of the sensors, which are primarily responsible for abnormal behaviors (anomalies or faults).

**Figure 6:**
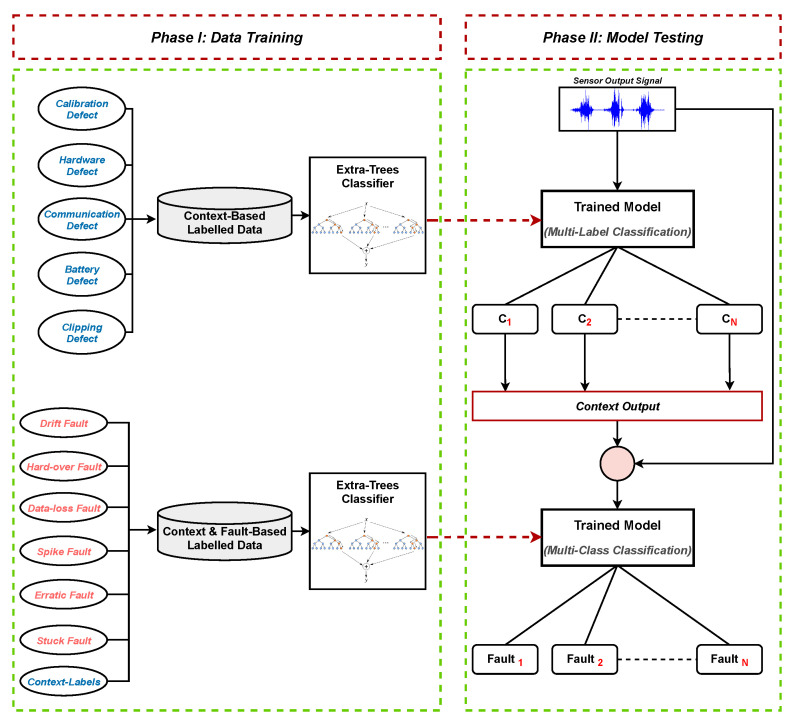
Framework of the proposed CAFD: the Context-Aware Fault Diagnostic scheme.

In Figure 4, the data-centric or soft faults (i.e., drift, hard-over, data-loss, spike, erratic, and stuck faults) are represented by lines to their context, respectively. The system-centric or hard defects (i.e., calibration, hardware, communication, battery, and clipping defects) that can be declared as contexts of the sensors are the prime causes of data-centric faults. The framework of the proposed CAFD system is presented in Figure 6.

Following data acquisition and preparation, the data samples are given as input to the ML classifier for training. Each data sample is labeled according to the corresponding context. For instance, hard-over fault samples are labeled 1 for calibration, and 0 for the rest of the contexts, since a hard-over fault substantially transpires due to calibration. Table 2 lists the labels for each class under consideration in this work.

Furthermore, the context-based multi-label data are first trained using the ET algorithm for classification purposes. Subsequently, the context-based classifier (ET) is given distinct sensor output signals (or data samples) to identify. This technique classifies each sample according to its context $\left(C_{1},C_{2},\ldots C_{n}\right)$, which belongs to any one of the aforementioned contexts, such as calibration.

Afterwards, the output of the context-based classifier in the form of labels is utilized as input features in the fault-based classifier. The fault-based multi-class data contain legitimate and faulty data samples along with the additional features from the context-based classifier. Furthermore, the fault-based multi-class data, labeled with normal and data-centric faults classes as mentioned above, are used to train the ET classifier.

To detect and diagnose data-centric faults, the fault-based classifier is given the sensor output signals $S_{n}$ in the form of data observations. The final classification is performed by the fault-based classifier (ET), which leads to the diagnosis of faults in the sensors.

## 5. Simulations and Results

### 5.1. Data Acquisition and Preparation

To evaluate the performance of ML classifiers, the data under consideration play an essential role. It is ideal to get data with genuine faults obtained from realistic scenarios. There are no publicly available datasets that in fact address all the faults in sensors. To conduct this research, we acquired a dataset published by the researchers at the University of North Carolina [32]. This dataset is composed of humidity and temperature sensor measurements. The data were acquired through Telos-B motes while creating single-hop and multi-hop WSN scenarios. For this research, we obtained the multi-hop data (only healthy measurements) and injected it with the six diverse sensor faults: drift, hard-over, spike, erratic, stuck, and data-loss. This approach is common among researchers needing to obtain faulty datasets [17,18,19].

**Figure 7:**
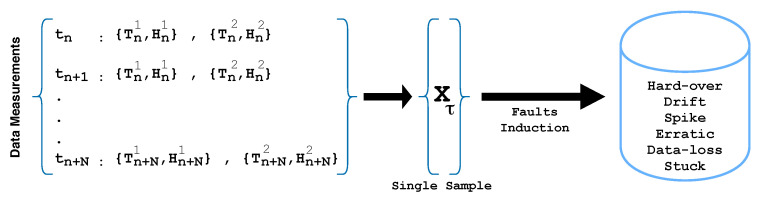
Illustration of the data preparation.

To prepare the dataset, we generated 16-dimensional data samples (measurements or vectors). Each sample was composed of 16 data points in 4 successive instances (t0,t1,t2,t3). Each instance was constructed from 2 humidity sensor measurements (H1,H2) and 2 temperature sensor measurements (T1,T2). In each instance, H1 and T1 measurements belonged to the first node, whereas H2 and T2 belonged to the second node of the multi-hop scenario. Figure 7 illustrates the data wrangling process.

Overall, 400×16 normal (legitimate/healthy) data points or observations were initiated. Afterwards, the six above-mentioned distinct sensor fault types were injected into the normal (non-faulty) data via simulations using Equations (2)–(6). To replicate a realistic scenario of a WSN, half of the data points were used to introduce faults in the first node, while the other half were used in the second node of the multi-hop network. Some of the faults (such as drift, hard-over, spike, and erratic) were induced with different intensities of fault (0.1,0.2,…,1.0), whereas, in the case of data-loss and stuck faults, the sensor’s output is either null or an unchanging constant value. The higher the fault intensity, the higher the fault rate in the data. For instance, the value 0.1 in the fault intensity corresponds to the lowest fault rate, whereas 1.0 is the highest. However, the data-loss and stuck fault samples remained unchanged throughout. Intuitively, the accuracy of the classifier improves with the increase in fault intensity.

Considering the normal class (or legitimate data) and the six above-mentioned faulty classes, the final dataset was composed of 7×400×16 data points. In each class, 60% of the data samples were used to train the ML classifier, whereas 40% were used for testing. In this work, two different labeling techniques were used to classify data (i.e., multi-class classification and multi-label classification), as explained in Section 4. In the multi-class technique, a single column of labels was introduced, while for the multi-label technique, five distinct columns of binary numbers were taken into consideration, according to the context of each class. Table 2 shows the labels in terms of numerical value for each class.

### 5.2. Results

To perform the simulations, the algorithms under consideration in this work were constructed in Python, utilizing the Scikit-learn and NumPy libraries. The Grid-Search Cross-Validation (CV) technique with CV=5 was used on the dataset to obtain the optimal hyperparameters for each algorithm to train. This technique works on the principles of *fit* and *score* in order to determine the best parameters, which can be used to train the ML models. In Table 3, these parameters are provided.

Generally, using a single performance evaluation metric for ML models is not considered good practice. In this work, three distinct metrics were taken into consideration to assess the performance of the classification algorithms. These metrics are defined as follows.

- **Diagnostic Accuracy** (DA) can be defined as the ratio of correctly diagnosed faulty or defective data samples to the total number of faulty samples.
  (7) $$DA=\frac{Number\;of\;faulty\;samples\;diagnosed}{Total\;number\;of\;faulty\;samples}$$
- **F1-score** or F-measure is described as the weighted average of precision and recall. This weighted average is commonly used to assess the performance of ML classification models.
  (8) $$F1-score=2\times \left(\frac{Recall\times Precision}{Recall+Precision}\right)$$
  where
  (9) $$Recall=\frac{True\;Positives}{Actual\;Positives}\;$$
  (10) $$Precision=\frac{True\;Positives}{Predicted\;Positives}$$
- **Area value under the ROC curve (ROC-AUC)** is an evaluation metric that calculates a scalar value in the range [0,1]. This measure determines how accurately the ML classifier can distinguish between faulty and non-faulty data observations. An accurate classifier can have an ROC-AUC value up to 1.0.

In this work, the without-context (or traditional) approach is simply distinguished from the context-aware (CAFD) approach as the scheme where sensor output signals are given in their genuine state to the ML classifier without considering the additional features extracted through the multi-label classification technique. The context-aware approach is based on multi-class as well as multi-label classification technique as shown in the CAFD framework (Figure 6). Furthermore, fault intensity depicts the rate of fault injected into the datasets. For instance, a 0.1 fault intensity corresponds to the lowest fault rate, whereas 1.0 is the highest. As the fault intensity increases, the performance of the classifier is also expected to improve. However, data-loss and stuck faults are free from the level of intensity due to the fact that sensor output is an unchanging constant value in both cases.

ET performance is shown in Table 4 in terms of F1-score for normal and distinct fault classes. The context-aware approach showed the ability to precisely distinguish between the maximum number of classes, compared to the without-context approach. The F1-score is increased overall by **9.28%** using a context-aware approach. Nevertheless, the drift, erratic, and data-loss fault classes have the same F1-score on both approaches. This is due to the reason that data points of these classes have a very unique structure compared to other classes, which makes it easy for any ML approach to classify. For instance, in the case of drift fault, the data sample values are linearly increased over time, while in an erratic fault, the data sample has high positive and negative values. Moreover, the data-loss fault sample is comprised of null values.

**Figure 8:**
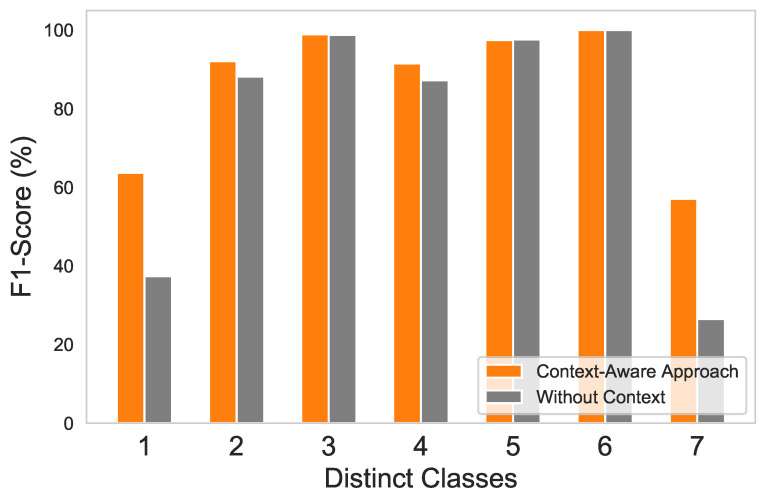
ET average F1-score comparison for distinctive classes using context-aware approach vs. without-context approach.

Furthermore, the normal class and the stuck fault class have highly identical data points, since sensor output undergoes low variation, which makes it hard for the classifier to discriminate between both situations and hence resulting in a low F1-score. The bar graph in Figure 8 explicitly provides the performance differences between the two approaches in terms of F1-score average. Each number in the graph depicts a different class: (1) normal, (2) hard-over fault, (3) drift fault, (4) spike fault, (5) erratic fault, (6) data-loss fault, and (7) stuck fault. While some of the classes have shown a somewhat similar F1-score to both approaches, most of them were improved with the context-aware approach.

**Figure 9:**
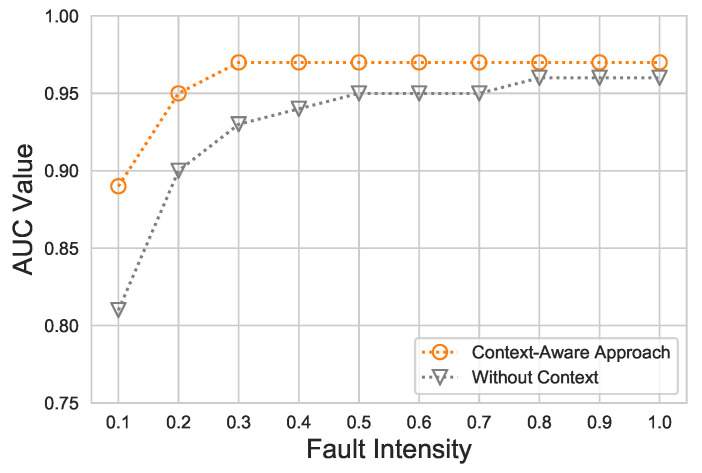
ROC-AUC against diverse fault intensities of the proposed ET-based context-aware approach, compared to the without-context approach.

In Figure 9, the ROC-AUC for ET versus different fault intensities under the proposed scheme is revealed. Starting from the lowest fault intensity, 0.1 up to 0.3, the ET-based context-aware approach AUC value increased considerably. The lowest AUC value noticed was 0.89. However, from 0.3 to 1.0, the proposed approach constantly achieved the maximum AUC value, up to 0.97. On the other hand, the same classifier (ET) in the without-context approach with hyperparameters identical to those in the context-aware approach revealed low performance, up to 0.81, whereas with the increase in fault intensity, the ROC-AUC was also elevated.

**Figure 10:**
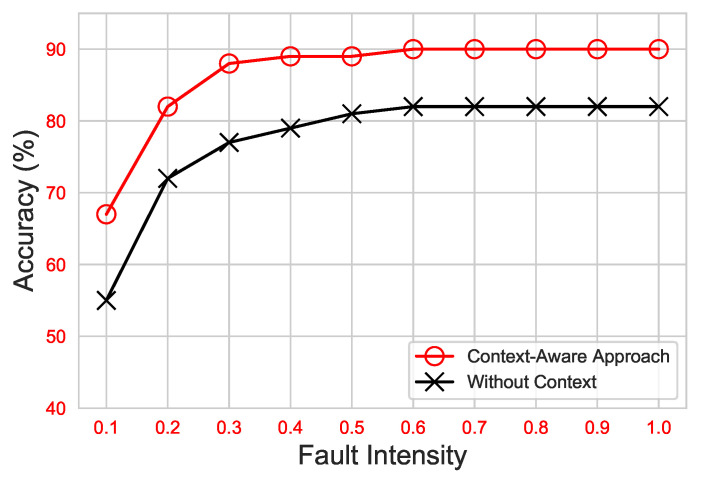
Diagnostic accuracy of the context-aware approach vs. the without-context (traditional) approach for ET on diverse fault intensities.

Figure 10 shows the average DA of the approaches under consideration. As can be seen, both approaches significantly improved with the increase in fault intensity. Nonetheless, utilizing the ET classifier, the proposed context-aware approach achieved a maximum accuracy of up to 90%, whereas the without-context approach had the utmost DA, up to 81%.

**Figure 11:**
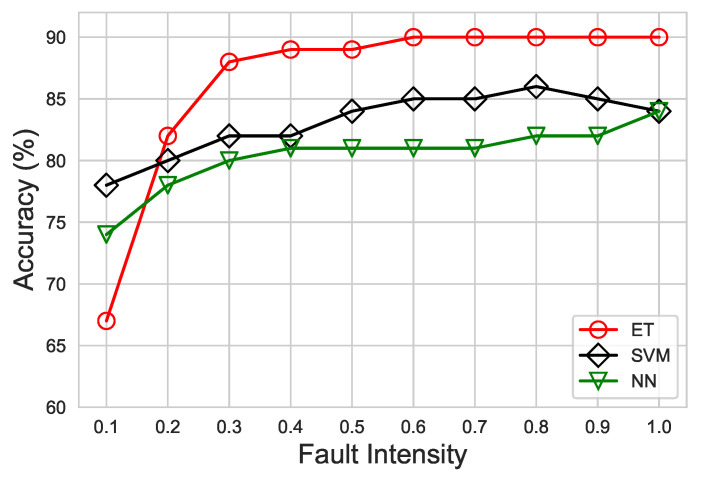
Diagnostic accuracy comparison of ET versus SVM and NN under the proposed CAFD scheme.

Furthermore, the DA of ET, SVM, and NN classifier at different fault intensities are shown in Figure 11. As the intensity of fault decreases, the disparity between normal and faulty data points reduces, so the performance of algorithms also diminishes. Moreover, an abrupt drop in accuracy for ET can be noticed at a fault intensity of 0.1. This is due to the reason that ET retreats data observations without replacement and uses a random splitting technique while training. This random nature of ET during training causes deterioration at an intensity of 1.0. Nevertheless, this limitation can be easily overcome by increasing a small amount of data observations at low intensity if needed. Overall, ET has attained the highest DA, up to 90%, compared to SVM and NN. This observation reveals that ET can achieve high classification accuracy for a multiple class problem by significantly reducing bias/variance error.

**Figure 12:**
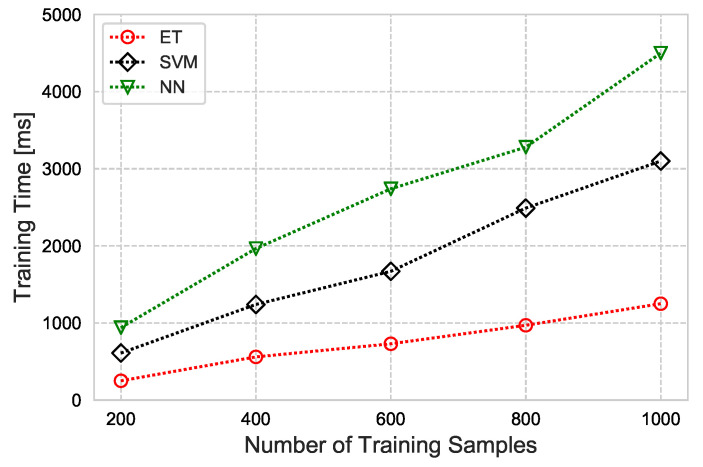
Training time based on the number of training samples for ET, SVM, and NN.

Finally, the time taken by each classifier to train, based on the number of training samples, is illustrated in Figure 12. We observed that ET is computationally inexpensive, compared to the SVM and NN. It is easy to state that utilizing ET for lightweight systems under the proposed CAFD scheme can achieve high performance by precisely detecting and diagnosing sensor faults.

## 6. Conclusions and Future Work

In this work, a lightweight CAFD scheme is proposed for timely detection and diagnosis of low-intensity faults in sensors. First, a dataset composed of healthy humidity and temperature sensor observations was acquired. Afterwards, the faults commonly occurring in sensors (drift, hard-over/bias, erratic/precision degradation, spike, stuck, and data-loss) were injected at different intensities into the dataset to generate a realistic defective WSN scenario. Healthy and faulty data observations were labeled utilizing multi-label/multi-class classification techniques for experimental purposes. These data observations were then used to train ML classifiers. The proposed CAFD scheme is composed of two ML-based classifiers: the first classifier carries out the multi-label classification task and the second classifier performs the multi-class classification task. Using multi-label classification, each fault was labeled according to its possible causes or context, such as calibration, hardware, communication, battery, and clipping. This context-based labeled data were then trained by an ensemble-method-based ML classifier named ET to diagnose the causes of each fault. Subsequently, the output of the ET classifier was used as additional features in fault-based labeled data to train the second ET classifier, which diagnosed the sensor faults such as drift. Extensive simulations revealed that using the contexts of the sensors as additional features in the original data observations can significantly improve a classifier’s performance. Furthermore, the proposed ET-based classifier in the CAFD scheme showed more efficiency than the SVM and NN in terms of diagnostic accuracy and training time.

In future work, we aim to increase the number of sensors in the network to check the robustness of the proposed scheme. Other than that, in the case of diagnostic accuracy, the performance of the proposed scheme was slightly deteriorated at the lowest intensity of fault, which emphasizes the need for further improvement in future work that could result in superior performance.

## Figures and Tables

**Table 1:** List of acronyms and abbreviations.

AI	Artificial Intelligence
CAFD	Context-Aware Fault Diagnostic
CNN	Convolutional Neural Network
CPS	Cyber–Physical System
CV	Cross-Validation
DA	Diagnostic Accuracy
DL	Deep Learning
DT	Decision Tree
ET	Extra-Trees
FDNN	Fuzzy Deep Neural Network
IoT	Internet-of-Things
ML	Machine Learning
NN	Neural Network
ROC-AUC	Area under the ROC curve
RF	Random Forest
SVM	Support Vector Machine
WSN	Wireless Sensor Network

**Table 2:** Representation of labels for each class according to the context.

Label	Class	Calibration Defect	Hardware Defect	Communication Defect	Battery Defect	Clipping Defect
1	Normal/Legitimate	0	0	0	0	0
2	Hard-over Fault	**1**	0	0	0	0
3	Drift Fault	**1**	0	0	0	0
4	Spike Fault	0	**1**	**1**	**1**	0
5	Erratic Fault	0	0	0	**1**	0
6	Data-loss Fault	**1**	**1**	0	0	0
7	Stuck Fault	0	**1**	**1**	**1**	**1**

**Table 3:** Parameters acquired by the grid-search approach for training the algorithms.

Algorithm	Parameters
Extra-Trees	n_estimators = 150 max_features = auto min_samples_split = 2 criterion = gini
Support Vector Machine	kernel = poly decision_function_shape = ovr gamma = auto C = 1.0
Multi-Layer Perceptron	hidden_layer_sizes = 100 max_iter = 1000 solver = lbfgs activation = identity learning_rate = constant

**Table 4:** ET F1-score comparison of individual classes on context-aware approach vs. without-context approach.

Class	*Context-Aware Approach*	*Without-Context Approach*
	F1-Score	F1-Score
Normal/Legitimate	63%	37%
Hard-over Fault	92%	88%
Drift Fault	98%	98%
Spike Fault	91%	87%
Erratic Fault	97%	97%
Data-loss Fault	100%	100%
Stuck Fault	57%	26%
	Average: **85.42%**	Average: **76.14%**

## Data Availability

No data available.
